# Application of 3D printing technology for pre-operative evaluation, education and informed consent in pediatric retroperitoneal tumors

**DOI:** 10.1038/s41598-023-28423-4

**Published:** 2023-01-30

**Authors:** Joong Kee Youn, Sang Joon Park, Young-Hun Choi, Ji-Won Han, Dayoung Ko, Jeik Byun, Hee-Beom Yang, Hyun-Young Kim

**Affiliations:** 1 Department of Pediatric Surgery, Seoul National University Hospital, Seoul, Korea; 2 Department of Pediatric Surgery, Seoul National University College of Medicine, 101 Daehak-ro, Jongro-gu, Seoul, 03080 Korea; 3 Department of Radiology, Seoul National University Hospital, Seoul, Korea; 4 Department of Surgery, Seoul National University Bundang Hospital, Gyunggi, Korea

**Keywords:** Paediatric research, Gastrointestinal system

## Abstract

To investigate usefulness of 3D printing for preoperative evaluations, student and resident education, and communication with parents or guardians of patients with pediatric retroperitoneal tumors. Ten patients planning retroperitoneal tumor resection between March and November 2019 were included. Preoperative computed tomography (CT) images were used for 3D reconstruction and printing. Surveyed items were understanding of preoperative lesions with 3 different modules (CT, 3D reconstruction, and 3D printing) by students, residents, and specialists; satisfaction of specialists; and comprehension by guardians after preoperative explanations with each module. The median age at operation was 4.2 years (range, 1.8–18.1), and 8 patients were diagnosed with neuroblastoma. The 3D printing was the most understandable module for all groups (for students, residents, and specialists, *P* = 0.002, 0.027, 0.013, respectively). No significant intraoperative adverse events or immediate postoperative complications occurred. All specialists stated that 3D printing enhanced their understanding of cases. Guardians answered that 3D printing were the easiest to comprehend among the 3 modules (*P* = 0.007). Use of 3D printing in treatment of pediatric patients with retroperitoneal tumors was useful for preoperative planning, education, and parental explaining with obtaining informed consents.

## Introduction

3D printing has expanded its medical applications in a variety of fields, including education, preoperative treatment planning, and custom instrumentation^1^. In application for pediatric patients, 3D printing has been mainly used to plan treatments for congenital diseases^2–4^. Use of 3D printing prior to resection of a complex pediatric abdominal tumors could be helpful for surgical treatment without complications^5^. However, the application development is still at an early stage, and few studies have been conducted using pediatric patient models^6,7^.

Tumors in a child's retroperitoneal cavity, especially in the cases of neuroblastoma, invade the surrounding structures, which is unexpected from pre-operative 2-dimensional abdominal computed tomography (CT)^8,9^. Pediatric surgeons have wanted to develop 3-dimentional tools for better surgical planning for this reason, which could result in better surgical outcomes by avoiding incomplete excisions or unexpected vascular injury^5–8^. It is also difficult for medical students and surgical residents to understand the anatomical location of the retroperitoneal tumors and how they should be operated on^10^. Therefore, there has been a demand for tools for intuitively visualizing and teaching anatomical structures. 3D printing can be applied to meet these needs because utilization of 3D printing in anatomical education could be a bridge of the large gap in experience by improving spatial comprehension of complex anatomy topics in a format that is widely accessible to most audiences^11^. In addition, caregivers are even less likely to understand lesions and surgery when they are explained by two-dimensional imaging alone^12,13^.

We performed 3D printing prior to pediatric retroperitoneal tumor resection to determine whether 3D printed models are helpful for preoperative planning by pediatric surgeons, education of students and surgical residents, and parental explanations during acquisitions of informed consent.

## Methods

### Inclusion criteria

Patients under 18 years of age who underwent retroperitoneal tumor resection at Seoul National University Children’s Hospital from March to November in 2019 were included. This study was conducted after obtaining consents from the participants. Patients who did not agree to participate in the study were excluded. We received approval for the study from Seoul National University Hospital (SNUH)’s Institutional Review Board (No. 2003-135-1110). This research was performed in accordance with the relevant guidelines and regulations. Informed consent was obtained from all participants’ parents or legal guardians.

### Research procedure and survey items

Imaging modules for study were prepared as follows; Enrolled patients underwent abdominal CT imaging routinely for preoperative planning. 3D reconstruction images were created based on the recent CT images, and 3D printed models were made based on them.

In order to investigate the understanding of the lesions for preoperative planning, 3 groups of 3rd grade medical students (N = 30, N = 10 for each module), residents (N = 30, N = 10 for each module), and certified pediatric surgeons (N = 5) reviewed lesions and major structures for each patient using 2D CT, 3D reconstruction, and 3D printing modules (Table 1). For each patients, three participants in each group were assigned to each module independently for evaluation. Margin of the mass, location of the structures, easiness of identification and degree of satisfaction were evaluated on a 0 to 5 point scale, with 0 meaning poor and 5 meaning excellent. After an evaluation of comprehension, the results of each participant group were compared among modules. In addition, we also investigated the opinions of the pediatric surgeons actually performing the surgeries, the difficulty of finding the structure in the actual surgical field using the three modules, and the overall satisfaction with 3D printed models. In addition, in order to confirm the understanding of the caregivers, the lesions were explained before the surgery by 2D CT, 3D reconstruction, and 3D printing in succession, and the understanding added to previous modules was checked. The survey items are summarized in Table 2.

**Table 1:** Demographics of students, residents and certified pediatric surgeons.

	Data
Students	N = 30
Male (%)	21 (70.0)
Female (%)	9 (30.0)
Grade (%)	
1st	0
2nd	0
3rd	30 (100)
4th	0
Residents	N = 30
Male (%)	18 (60.0)
Female (%)	12 (40.0)
Years (%)	
1st	8 (26.7)
2nd	7 (23.3)
3rd	5 (16.7)
4th	10 (33.3)
Certified pediatric surgeons	N = 5
Male (%)	2 (40.0)
Female (%)	3 (60.0)
Years of experience (years)	5 (1–17)

**Table 2:** Survey items.

Preoperative evaluation of comprehension (for students, residents and certified pediatric surgeons)
1. Mark margin and borders of the mass
2. Mark running of Lt./Rt. ureter
3. Mark running of Lt./Rt. renal artery
4. Mark running of Lt./Rt. renal vein
5. Mark running of celiac axis
6. Mark running of superior mesenteric artery
7. Mark running of splenic vein
8. Estimate size of the mass
Evaluation of conclusive comprehension and satisfaction after operations (for certified pediatric surgeons)
1. Easiness of identification between the mass and surrounding objects by CT
2. Easiness of identification between the mass and surrounding objects by 3D reconstruction
3. Easiness of identification between the mass and surrounding objects by 3D printing
4. Degree of satisfaction about the expression between the mass and surrounding objects by 3D printing
5. Easiness of operation by applying 3D printing
6. Overall satisfaction for the whole treatment process using 3D printing
Evaluation of understanding for parents/ legal guardians
1. Degree of understanding of the lesion with CT
2. Degree of understanding of the lesion with 3D reconstruction
3. Degree of understanding of the lesion with 3D printing

CT, computed tomography.

In addition, we checked the operation time, estimated blood loss, number of adverse events during surgery, and complication and mortality occurring within 2 weeks after surgery.

### 3D printing model making process

Preoperative CT imaging with slice thickness of 3 mm is performed and sent to Medical IP^©^, a 3D printing agency. Medical IP^©^ uses MEDIP software platform (http://medicalip.com/Medip, MEDICAL IP^©^, Seoul, Republic of Korea) for medical image processing, which applies machine learning-based thresholds, region growing, and graph-cut algorithms (Supplementary Figure). The graph-cut algorithm is an algorithm that separates the foreground from the background by configuring each pixel with graph intersections (nodes) and utilizing the difference in energy (flow network) between them^14^. In order to reconstruct CT images in three dimensions, segmentation of each organ is performed and verified by pediatric radiologists to confirm their adequacy. The platform is equipped with a security device for protecting personal information, so that researchers related to image handling can check and feedback only through the 3D viewing platform through a security pledge process.

**Figure 1:**
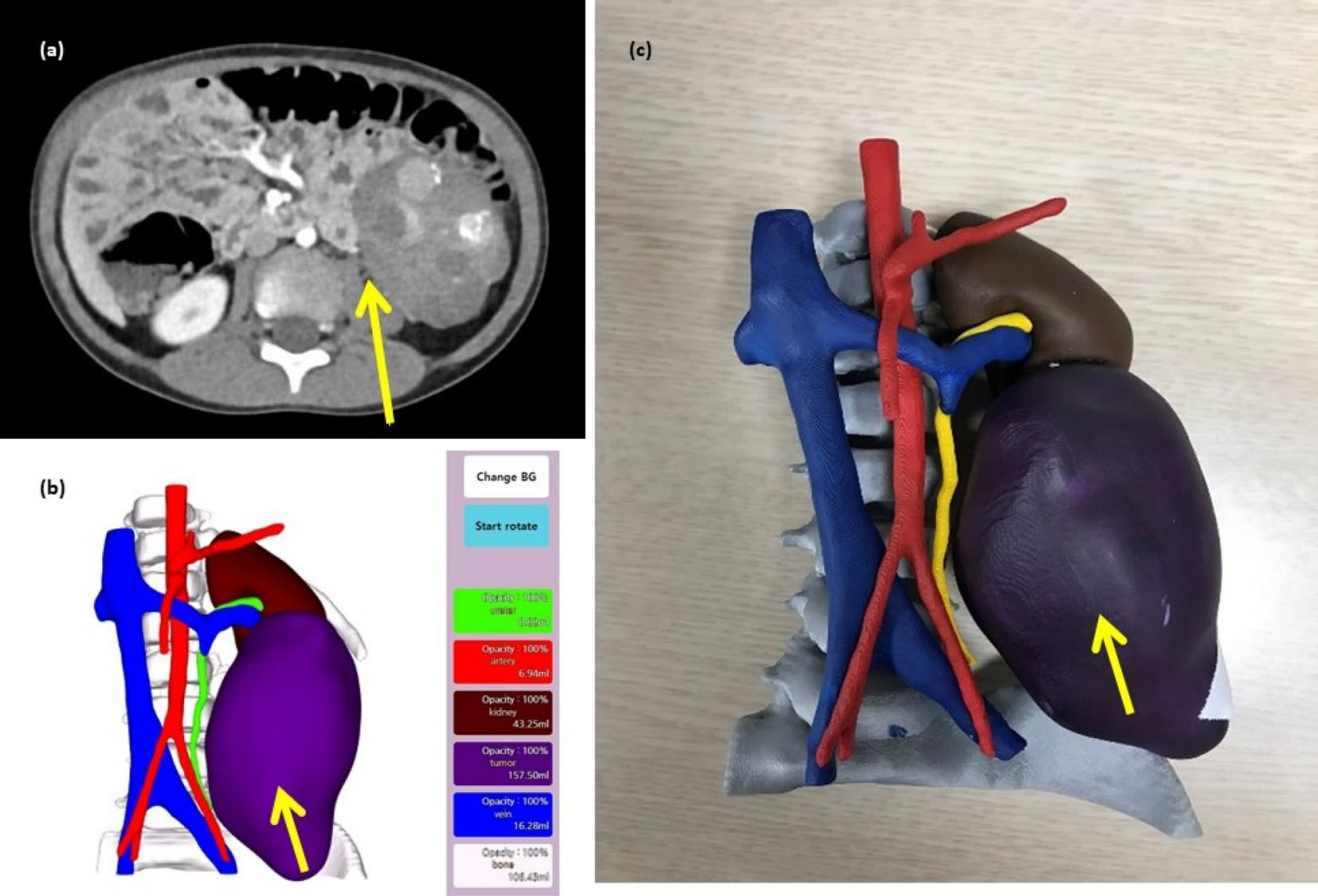
Computed tomography scan of a 4-year-old male patient with neuroblastoma (**a**), 3D reconstruction (**b**), and 3D printed model.

After confirming the 3D reconstructed image and reviewing the medical information by the clinicians, the 3D printing model was manufactured. Models were produced on a 1:1 scale, and printed with acrylonitrile butadiene styrene (ABS) copolymers (400–500 g per case) and resin (800–1200 g per case) by the Polyjet method (J750 [Stratasys; 7665 Commerce Way Eden Prairie, MN 55344, USA]). The 3D printing model was completed at least 3 days before surgery, allowing the medical staff to identify the case and use it for explanations to the caregiver (Fig. 1).

### Statistical analysis

Categorical variables are presented as percentages. Comparisons between categorical variables of three groups were performed with the Kruskal–Wallis due to the non-parametric nature of the data followed by the post hoc Bonferroni Correction. Continuous variables are reported as the median with range or the mean ± standard deviation. A *P* value < 0.05 was considered significant in Kruskal–Wallis test and < 0.0167 in the post hoc analysis. The IBM SPSS Statistics 20.0 statistical software package (SPSS Inc., Chicago, Illinois, USA) was used for all statistical analysis.

## Results

### Data related to surgical information

Ten patients were included during the study, 8 of which were boys. Median age was 4.1 (range, 1.8–18.1) years and mass size was 2.1 (range, 1.0–10.9) cm. Eight of the ten patients were diagnosed with neuroblastoma. There were no unexpected injury or amputation of blood vessels and ureters during surgery, and there was no post-operative of complications and mortality (Table 3). The demographic and operation-related information of each patient is listed in the Table 4.

**Table 3:** Demographics and operation-related data.

	Patients (N = 10)
Male (%)	8 (80.0)
Age at operation (years)	4.1 (1.8–18.1)
Size of mass (cm)	2.1 (1.0–10.9)
Diagnosis (%)	
Neuroblastoma/Ganglioneuroblastoma	8 (80.0)
Ganglioneuroma	1 (10.0)
Extralobar pulmonary sequestration	1 (10.0)
Intra-operative events	
Estimated blood loss (mL)	148.0 ± 124.5
Unexpected sacrifice of major vessels and ureter (%)	0
Operation time (min)	207.5 ± 95.2
Postoperative complications/mortality (%)	0

**Table 4:** Detailed information of enrolled patients.

No	Sex	Age at op (years)	Bwt at op (kg)	Diagnosis	Name of operation	Size of mass (cm)	Underlying conditions	EBL (mL)	Op time (min)
1	M	4.6	16.2	GNBL	Lap. retroperitoneal tumor excision	9.6	None	60	235
2	M	1.8	11	ELS	Laparoscopic ELS excision	2.0	None	0	65
3	M	4.7	15.6	GN	Lap. retroperitoneal tumor excision	1.1	None	20	80
4	F	4.2	17.4	NBL*	Intra-abdominal metastatic LN excision	1.0	None	120	300
5	M	18.1	50.2	NBL	Pelvic mass excision	2.2	HS	300	175
6	M	4.0	16.6	NBL	Retroperitoneal tumor excision	3.8	None	150	275
7	M	2.3	12.5	NBL	Lap. retroperitoneal tumor excision	6.4	None	140	200
8	M	3.8	15.5	NBL	Left para-aortic mass excision	1.7	None	200	150
9	F	13.7	52.2	NBL	Lap. retroperitoneal tumor excision	10.9	ARM	400	370
10	F	2.0	12.2	NBL*	Intra-abdominal metastatic LN excision	4.0	None	90	225

Op, operation; EBL, estimated blood loss; GNBL, ganglioneuroblastoma; ELS; extralobar pulmonary sequestration; GN, ganglioneuroma; NBL, neuroblastoma; *: Mass with intra-abdominal metastasis, LN: Lymph nodes, HS: Hereditary spherocytosis, ARM: Anorectal malformation (Intra-abdominal metastatic LN excision: subhepatic, common ilac, internal iliac, paraduodenal, and mesentery LN excision).

**Figure 2:**
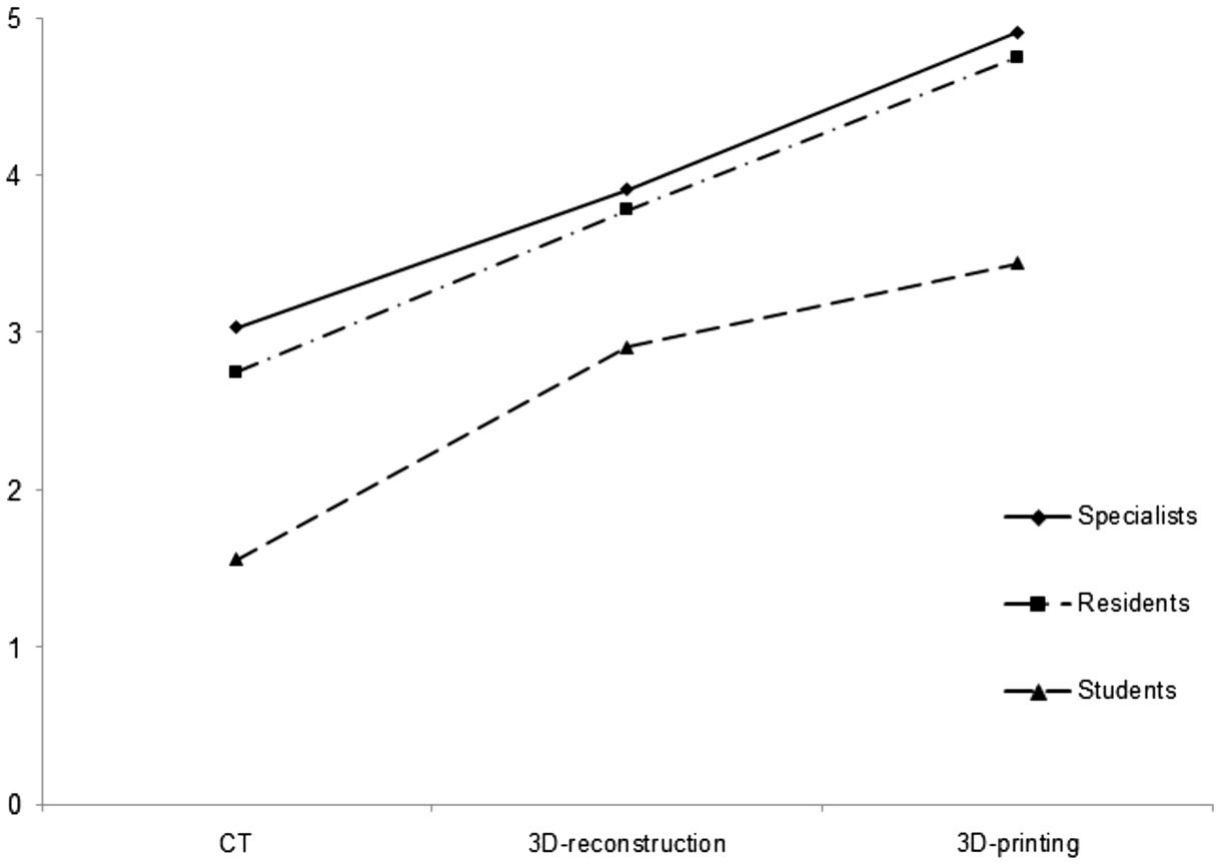
The understanding of lesions using computed tomography, 3D reconstruction and 3D printed models by each group.

In all 3 groups (students, residents, and certified pediatric surgeons), the understanding of lesions using 3D printed models was the highest, and the comprehension was lowest using CT, which was statistically significant in each group (*P* = 0.005, 0.027, 0.013, respectively) (Fig. 2, Table 5). Post hoc analysis found statistical differences between CT vs 3D reconstruction and CT vs 3D printing (P < 0.001 for each), and no difference between 3D reconstruction and 3D printing in all three groups.

**Table 5:** Degree of preoperative comprehension by using CT, 3D reconstruction, and 3D printing model by certified pediatric surgeons, residents and students.

	CT	3D reconstruction	3D printing	*P* value
Pediatric surgeons	3.03 ± 0.53	3.91 ± 0.43	4.91 ± 0.12	0.002
Residents	2.75 ± 0.35	3.78 ± 0.37	4.75 ± 0.17	0.027
Students	1.56 ± 0.29	2.91 ± 0.39	3.44 ± 0.50	0.013

Scale: 0 (poor) to 5 (excellent).

CT, computed tomography.

When the students performed pre-operative evaluation through CT, 6 out of 10 did not indicate the mass margin at all in the comprehension question. Only 2 were able to indicate the flow of ureter and renal artery/vein more than 50%. In the case of using 3D reconstruction, 7 out of 10 patients indicated a mass margin of 50% or more, and this number increased to 9 in 3D printing. When 3D reconstruction and 3D printing were used, 5 and 8 patients displayed more than 50% of renal vessels, respectively. A similar trend was also observed in residents. In the pediatric surgeon group, even when only CT was used, margin of the mass was recognized by more than 50% in 6 cases. The number increased when 3D reconstruction and 3D printing were used as in the case of students and residents, and similarly observed for blood vessel recognition.

Only the certified pediatric surgeons evaluated the difference between each module and the actual surgical field; it was found that it was the easiest to identify the field through 3D printed models and it was statistically significant (*P* = 0.018). In the comparison between each group, there was no significant difference between CT and 3D reconstruction, 3D reconstruction and 3D printing, and only CT and 3D printing showed a significant difference (*P* = 0.005). Overall satisfaction with the 3D printing module was found to be 4.3(± 0.3) out of 5 points (Table 6).

**Table 6:** Conclusive comprehension and satisfaction by certified pediatric surgeons.

	CT	3D reconstruction	3D printing	*P* value
Degree of identification easiness	3.71 ± 0.95	4.28 ± 0.76	5.0 ± 0.0	0.018
Overall satisfaction	–	–	4.3	–

Scale: 0 (poor) to 5 (excellent).

CT, computed tomography.

### Data related to informed consent

Two out of 10 caregivers were male; 7 of them were in their 30 s. Nine of them had a higher education than being college graduates (Table 7). The comprehension by the guardians was measured after using CT only, CT and 3D reconstruction, and all three modules including 3D printing, and their understanding scores were 2.71(± 1.25), 4.29(± 0.95), and 4.86(± 0.38) out of 5, respectively, with *P* value as 0.007.

**Table 7:** Demographics and survey results on parents/legal guardians.

	Parents/legal guardians (N = 10)
Male (%)	2 (20.0)
Age at survey (%)	
< 20	0
20–29	0
30–39	7 (70.0)
40–49	3 (30.0)
≥ 50	0
Educational degree (%)	
High school	1 (10.0)
College	6 (60.0)
Graduate school	3 (30.0)
Degree of understanding (0–5)*	*P* = 0.007
CT	2.71 ± 1.25
3D reconstruction	4.29 ± 0.95
3D printing	4.86 ± 0.38

CT, computed tomography.

*Scale: 0 (poor) to 5 (excellent).

## Discussion

This study was the first case series to prospectively apply 3D printing to treatment of pediatric tumor patients. Preoperative planning using 3D printed models increased the operator's satisfaction and adverse events during surgery occurred only in very few cases. Its use in educating students and residents was found to be highly effective, and the satisfaction of treatment was improved by better understanding when acquiring pre-operational consent for guardians.

A study of preoperative planning, with a simple choledochal cyst model using polymer powders, reported that 3D printing could be useful for preoperative simulation^2^. In patients needing complex surgery, preoperative planning using 3D printing takes less time and has better accuracy for identifying structures than CT or 3D reconstruction^15^. In a study by Krauel et al.^5^, who performed 3D printing on 3 pediatric oncologic cases, the use of 3D printing could improve pre-tumor planning in a variety of fields by increasing the extent and safety of resection. In our study, as in previous studies, unexpected events during surgery and postoperative complications were not observed. It's not entirely due to the help of 3D printing, but being able to get more information when planning preoperatively may be one of the reasons why it has fewer adverse outcomes. In particular, this was also observed by the records of patients 4 and 10. They had excisions of intra-abdominal metastatic lymph nodes of various locations such as para-duodenal, para-aortic, retrorenal and portocaval areas and recorded a lower-than-average expected blood loss despite a higher risk of damage to nearby blood vessels due to previous operations and the location of the lymph nodes. This could be inferred from the operator's high satisfaction with the preoperative evaluation.

Several studies have reported the results of using 3D printing in the education of students and residents. A recent study reported on 3D printing models with a variety of colors to replace the anatomy education with cadaveres^16,17^, and 3D-printed liver was reported to be more efficient than a traditional atlas in anatomy education^18^. In education of pathologic models of aortic aneurysm and cancers, 3D printing was useful for surgical education from the survey results^19^. Hojo et al.^20^ reported a model of lateral pelvic lymph node dissection for training purposes, and showed that 3D printing was superior to textbooks for learning pelvic anatomy. In a recently reported meta-analysis, it was reported that education using 3D printing was more helpful in improving students' understanding^11^. In our study, students and residents compared 2D CT, 3D reconstruction, and 3D printing for understanding retroperitoneal tumors. Similar to previous studies, the 3D printing enabled the highest level of understanding.

Personalized 3D printing has the potential to improve patient comprehension in informed consent for surgical resection in patients suspected of having stage I lung cancer^13^. Yang et al.^12^ also reported the using 3D printed liver models improved parental education regarding the understanding of liver anatomy and physiology, tumor characteristics, surgical procedure, and associated surgical risks. As reported in the previous studies, we found that the informed consent obtained using 3D printed models in addition to the conventional 2D CT images improved the understanding and satisfaction of the caregivers in the management of retroperitoneal mass resection.

This study is an initial pilot study involving only 10 patients with retroperitoneal tumors, and has a limitation in that it has a small number of case series. More patients need to be enrolled for evaluation of the roles of 3D printing in the management of pediatric patients. In our report, the accuracy of 3D printing was good enough that operators reported high satisfaction with the 3D printing models, but more attention should be paid to avoid possible deviations as warned in Martelli et al.'s review paper^21^. In the future, our center will conduct 3D printing of various pediatric congenital diseases, as well as retroperitoneal tumors for preoperative planning, to verify the effectiveness in educating students in normal and pathologic anatomy.

## Conclusion

The use of 3D printing in pediatric patients with retroperitoneal tumors can improve the understanding of pediatric surgeons. Education with 3D printing was helpful for understanding the lesion by students and residents; it also helped guardians to comprehend better in informed consents.

## Supplementary Information

Supplementary Information.

## Data Availability

The datasets used and/or analyzed during the current study available from the corresponding author on reasonable request.
