# Supplementary figures and images for: Application of 3D printing technology for pre-operative evaluation, education and informed consent in pediatric retroperitoneal tumors
Source: Sci Rep. 2023 Jan 30;13:1671. doi: 10.1038/s41598-023-28423-4 (PMC9886922; doi:10.1038/s41598-023-28423-4)

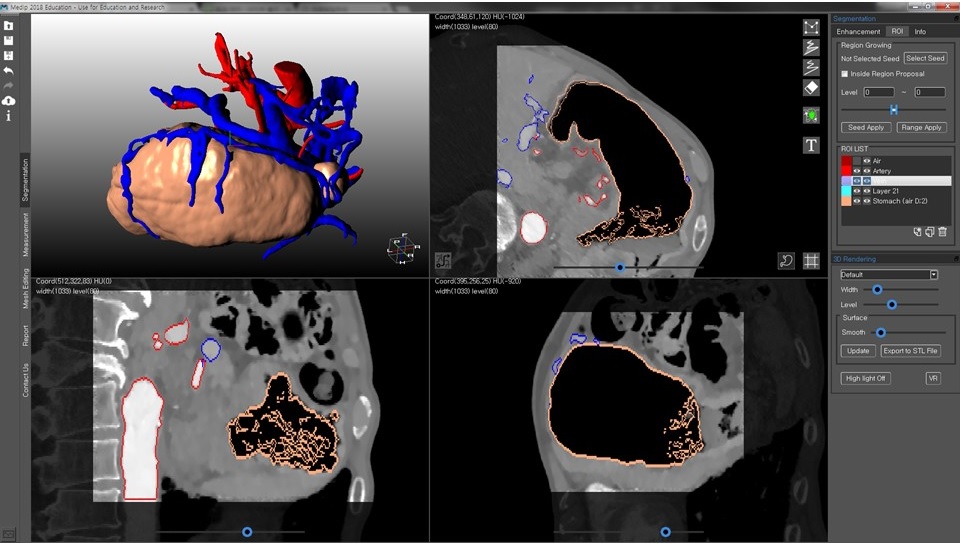

Supplement: Supplementary file 1 — Supplementary Information. [file 41598_2023_28423_MOESM1_ESM.jpg]
